# Recent Advances in Single Crystal Diamond Device Fabrication for Photonics, Sensing and Nanomechanics

**DOI:** 10.3390/mi12010036

**Published:** 2020-12-30

**Authors:** Dipti Rani, Oliver Roman Opaluch, Elke Neu

**Affiliations:** Fachbereich Physik, Technische Universität Kaiserslautern, Erwin-Schrödinger-Strasse, D-67663 Kaiserslautern, Germany; Diptidahiya@gmail.com (D.R.); opaluch@rhrk.uni-kl.de (O.R.O.)

**Keywords:** diamond, nanofabrication, photonics, quantum technologies

## Abstract

In the last two decades, the use of diamond as a material for applications in nanophotonics, optomechanics, quantum information, and sensors tremendously increased due to its outstanding mechanical properties, wide optical transparency, and biocompatibility. This has been possible owing to advances in methods for growth of high-quality single crystal diamond (SCD), nanofabrication methods and controlled incorporation of optically active point defects (e.g., nitrogen vacancy centers) in SCD. This paper reviews the recent advances in SCD nano-structuring methods for realization of micro- and nano-structures. Novel fabrication methods are discussed and the different nano-structures realized for a wide range of applications are summarized. Moreover, the methods for color center incorporation in SCD and surface treatment methods to enhance their properties are described. Challenges in the upscaling of SCD nano-structure fabrication, their commercial applications and future prospects are discussed.

## 1. Introduction 

Diamond is an outstanding material for micro/nanofabrication of optomechanical, photonic and sensor components owing to its exceptional mechanical properties, broad optical transparency combined with biocompatibility in both bulk and nano-structures. High quality, high purity single crystal diamond (SCD) is mainly grown via chemical vapor deposition (CVD) methods. SCD is mostly limited to sizes below 5 mm × 5 mm and a costly material. Only recently, SCD has become available in sizes up to 100 mm wafers via heteroepitaxy. Consequently, diamond based optomechanical and nanomechanical components are realized often on polycrystalline diamond films which can be easily grown on wafer scale. However, using polycrystalline material will limit the performance of photonic components and sensors due to light scattering, strain and defects [1]. With advancement in nanofabrication methods, various diamond structures have been realized including nanomechanical, nanophotonic and optomechanical systems. Nanomechanical systems, including nanoresonators, allow ultrahigh sensitivities in force detection and realizing optomechanical systems which couple optical and mechanical modes [2,3]. On the other hand, photonic components including nanopillar waveguides, photonic crystals (PhC), optical antennas, metalenses, solid immersion lenses (SIL) and other optical resonators are used for light manipulation, propagation, and trapping. Optically active point defects called color centers in diamond have proven useful for a wide range of applications which span from sensing magnetic fields e.g., in nano magnetic resonance, single molecule detection and single photon emission to optomechanics, etc. [3,4,5]. The most explored point defect in diamond, the nitrogen vacancy (NV) center provides an optically readable electron spin at room temperature even for individual color centers. However, photon collection efficiency from NV centers in bulk diamond is intrinsically limited due to the large refractive index of diamond (n ≈ 2.4) and total internal reflection at the diamond-air interface. Exceptional efforts were made to engineer high quality NV centers with long spin coherence times and realize diamond structures for efficient light extraction. Realizing such structures, often embedding single NV centers, requires growth and nanofabrication of high quality SCD. In recent years, many novel nano-structures have been realized for advanced sensing applications with NV centers replacing micro/nano devices developed earlier. However, NV center based devices are not perfectly suited for many quantum communication applications, as e.g., quantum repeaters, since their photon throughput is limited due to long fluorescence lifetime (≈11 ns) and weak emission into zero phonon line (ZPL, 4% at room temperature) [6]. Moreover, NV centers have a permanent dipole moment increasing their susceptibility to surrounding electrostatic fields and decreasing the rate of generation of indistinguishable photons [7]. 

Consequently, different types of color centers with emission ranging from visible to infrared are currently investigated. Among those, group-IV atoms based color centers such as silicon-vacancy (SiV), germanium-vacancy (GeV), tin-vacancy (SnV), and lead-vacancy (PbV) are worth mentioning which show narrow emission and promising coherence properties at low temperatures [6]. They possess inversion symmetry and thus are less susceptible to variations in electrostatic fields. The GeV and SnV centers are advantageous due to their larger energy splitting in the ground state which results in better coherent spin manipulation. GeV centers yield higher quantum efficiency (in comparison to SiV centers) and are thus favorable for applications in quantum optics and nanophotonics [7]. All these color centers show promising emission properties, novel wavelengths and have opened wide directions in scientific exploration. Extensive discussion about the creation and applications of color centers is beyond the scope of this review. 

Diamond nano-structures are developed using top-down and bottom-up approaches. Top-down approaches include SCD structuring from high quality bulk substrates (≈5 × 5 × (0.02–0.05) mm^3^) using state of the art lithography techniques and plasma etching methods. Impurity atoms forming color centers are introduced into SCD before or after nano-structuring often at precise locations via ion implantation. Within bottom-up approaches, diamond nano-structures are formed during diamond growth using CVD processes [8,9,10]. Defects are introduced via modifying the growth process (e.g., introduction of nitrogen) during CVD synthesis [3,11]. Bottom-up synthesis yields diamond nano-structures without the danger of inducing plasma etching induced defects. However, the density of color centers and shape/configuration of nano-structures is difficult to control via these methods. On the other hand, top-down approaches allow to create nano-structures with well-defined characteristics (shape, defect density) with high precision, but, require advanced nanofabrication and plasma etching which enhances processing costs [4,12]. 

In this review, we mainly focus on the advancement in top-down fabrication methods in the last 3 years for structuring of bulk diamond into various nano-structures, which has not been done recently. Complementary to the previous reviews by Schroeder et al. [13] and Aharonovich et al. [14], which reported mainly the fabrication of diamond photonic devices, we describe the different micro/nano-structures that were realized and are suitable for applications in quantum photonics, sensing, mechanics, optomechanics, etc. The surface modification methods developed for improvement of device properties are discussed. We also discuss challenges hindering mass production of such devices and possible solutions.

This review is organized as follows: Section 1 overviews the nano-structuring of diamond including incorporation of different color centers; Section 2 introduces the nanophotonic components and the methods deployed to realize different nanophotonic structures; Section 3 introduces the nanomechanical components and describes the different structures realized; Section 4 and Section 5 describe the developed optomechanical structures and other nano-structures, respectively; Section 6 summarizes the review and gives an outlook on how to improve diamond nano-structuring methods to upscale production and enhance reliability.

### Why Nano-Structuring of Diamond

Several commercial suppliers (e.g., Element 6 [15], Applied Diamond Inc. [16], AuDiaTec [17],) offer bulk diamond substrates of different size, quality, orientation, and defect density. Typically, substrates are characterized for their surface roughness and polishing wedge prior to nano-structuring. Structuring of diamond requires patterning of the resist mask using lithography techniques based on electron-beam (EBL), ultra-violet (UVL), nanoimprint (NIL) or laser or two photon lithography [18,19,20,21,22]. Owing to the high inertness of diamond, wet-etching methods are not suitable and thus transferring the pattern to the diamond is carried out using reactive ion etching (RIE). For etching of diamond, plasmas of special gases (oxygen/argon or chlorine-based mixtures) at high powers mostly in inductively coupled reactive ion etching (ICP-RIE) are used. Specific resists such as PMMA on hard masks layers (Al_2_O_3_, Al, W) or hydrogen silsesquioxane (HSQ) are typical material systems. 

Over the last decade, various nano-structures (nanopillars, nanowires, beams, nanogratings, etc.) were realized with NV centers incorporated via implantation of nitrogen atoms in high purity SCD. Although ion implantation precisely introduces dopants with optimal density and depth, it induces surface and lattice damage which affects the properties of the final devices. To overcome the limitation of ion implantation, an alternative approach includes high energy electron irradiation on ultrapure diamond to transform native nitrogen impurities into NV centers [23]. Nano-structures realized through such approaches are suitable for applications in quantum information processing, magnetometry, microscopy, nano magnetic resonance imaging, etc. [1,2,5]. 

## 2. Photonic Components

### 2.1. Basics of Photonic Components

Nanophotonic platforms are capable of interfacing optical elements (e.g., waveguides, lenses) with solid-state quantum emitters (e.g., luminescent color centers) and are widely explored in quantum technologies. In diamond based components for quantum information, photons interconnect spins associated with individual luminescent color centers (e.g., NV centers) thereby enabling entanglement between distant spins [24]. Quantum information is stored in the electron spins of NVs or nuclear spins of nearby atoms in the diamond whereas coherent state manipulation is achieved via applying microwave or RF fields. The information is communicated via encoding the spin information into degrees of freedom of the photons. However, only photons from the purely electronic transition namely the zero phonon line (ZPL) are usable in this context. Unfortunately, the emission spectrum of NV centers consists of ZPL and a broad phonon sideband with only 4% of emission into the ZPL. To boost usability of NV centers in quantum information, it is important to enhance emission into the ZPL using optical cavities [1,25]. In contrast, the non-resonant spin read-out used in room temperature sensing applications works also via the photons in the sideband and thus mostly concentrates on waveguide structures that allow for broadband collection enhancement. 

The main figure of merit for optical cavities is the Purcell factor (*P_F_*): It describes the enhancement in spontaneous emission rate for an optimally aligned dipole localized in the maximum of the cavity field:

(1) $$P_{F}=\frac{3}{4\pi^{2}}{\left(\frac{\lambda }{n}\right)}^{3}\;\frac{Q}{V}$$

where *V*, *n* and *λ* are the cavity mode volume, mode index and emission wavelength, respectively. The quality factor *Q* describes the storage time of photons in the cavity and depends upon the electric field decay rate in the cavity *κ* and the resonant frequency of the cavity *ω_c_*.

(2) $$Q=\frac{\omega_{c}}{2\kappa }$$

Thus, high quality factors (*Q*) and/or a low mode volume (*V*), resulting in long interaction times between the emitter and the photons and a strong electric field, are preferred. *Q*-factor and mode volume are used to characterize narrow bandwidth devices such as ring or disk resonators or photonic crystal resonators. In contrast, waveguide devices like nanopillars, lenses, gratings etc. will rather be characterized by their enhancement of the collection efficiency. 

Most photonic structures are not suitable for randomly distributed centers as a defined placement within the nano-structure is mandatory for optimal coupling [26]. Large Qs are easily obtained in near-infrared regime in large resonators such as ring-resonators and in devices with large cavities which can be easily fabricated. For photonic crystal cavities, 1-D cavities result in high *Q*-factors and *P_F_* owing to the small mode volumes, in comparison to 2-D photonic cavities with same parameters. The combination of optimal nanofabrication and precise positioning of color centers remains challenging as detailed below.

### 2.2. Fabrication of Thin SCD Membranes for Photonic Structures

**Figure 1:**
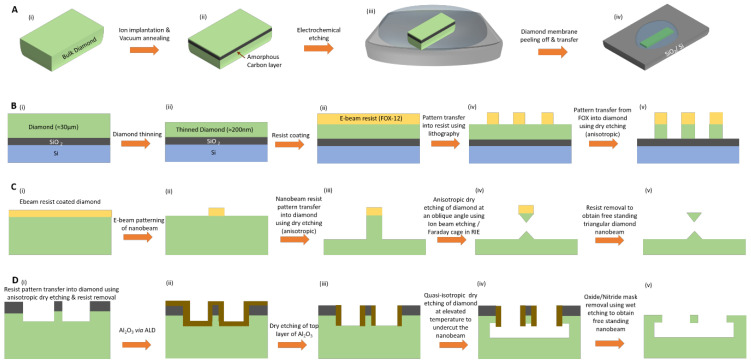
Schematics of different methods realized for diamond nanofabrication: (**A**) Lift-off method in which ions are implanted into bulk diamond to create an amorphous carbon layer (ii) few microns below the surface, followed by electrochemical etching of the damaged layer (iii) and transfer of the peeled-off diamond membrane over another substrate. Figures adapted from Ref. [27] originally published by The Royal Society of Chemistry under creative commons license CC BY 3.0; (**B**) Thin-down technique, where bulk diamond is transferred over another substrate (e.g., SiO_2_/Si) and dry etched to obtain thin diamond membranes of ≈200 nm (i,ii). The structures in nanomembrane are created by patterning of the spin coated resist using E-beam lithography (iii, iv), and subsequent transfer into diamond using dry etching process (v). Figures adapted from Ref. [28] copyright IOP publishing, Reproduced with permission. All rights reserved; (**C**) Angled etching method in which lithographically defined nanobeam resist pattern (i,ii) is transferred over the underlying diamond substrate using anisotropic dry etching process (iii), followed by dry etching at an oblique angle using ion beam etching or Faraday cage in RIE (iv) to release the diamond nano-structures and subsequent resist mask removal to obtain free-standing, triangular nanobeam (v). Figures reprinted (adapted) with permission from [33,39] Copyright (2019) by the American Physical Society for Ref. [33], for Ref. [39] reprinted under creative commons license CC BY 4.0; (**D**) Isotropic undercut etching method, where a combination of anisotropic and isotropic dry etching processes is used to obtain free-standing diamond nano-structures. Nanobeam pattern transfer using hard mask into diamond via a combination of e-beam lithography and anisotropic etching (i). The patterned diamond substrate is coated conformally with Al_2_O_3_ via ALD technique (ii) and dry etched (iii) to remove the top layer of Al_2_O_3_. Subsequently quasi-isotropic oxygen etching is carried out to undercut the nanobeam structure (iv) and wet etching of hard mask to obtain free-standing nanobeam structure over bulk diamond (v). Figures reprinted (adapted) from [34,36], for Ref. [34] with the permission of AIP Publishing; for Ref [36] under creative commons license CC BY 4.0.

Realization of photonic components often requires high quality, SCD membranes/structures of around few hundreds of nanometers which are difficult to grow in a controlled manner via bottom-up methods. Harnessing advanced nano-structuring methods for SCD, bulk SCD is structured into free standing, thin films (≈200 nm thickness) or free standing nano-structures. The deployed fabrication methods are briefly described in the following paragraphs:

- The lift-off method (illustrated schematically in Figure 1A) where SCD is implanted with ions to create a sacrificial layer below the SCD surface. The layer is graphitized in an annealing process (ii) and removed via electrochemical etching (iii). The thin, peeled-off SCD membrane is transferred to another substrate (iv) and pristine quality SCD is overgrown while the original, ion-damaged SCD membrane is etched away [27].
- The thin down technique in which commercially available, high-purity SCD (≈50 µm thick) is etched down to required thickness of few hundreds of nanometers via dry etching methods [28]. An example is illustrated in Figure 1B, thin SCD (≈30 µm) is transferred to SiO_2_/Si substrates for easy handling (i) and dry etched (ii) to obtain thin SCD membranes (≈200 nm). For nano-structuring, the diamond/SiO_2_/Si samples are spin coated (iii) and patterned via EBL (iv). The resist pattern is transferred into SCD using anisotropic dry etching processes (v), followed by resist mask removal.
- Direct cleavage method using a focused beam of ions such as Ga or O_2_ to directly cut (mill) SCD without any mask preparation. This method is limited in terms of scalability and the damage introduced due to implanted ions [29,30].
- Angle etching method which transfers a pattern defined using EBL into SCD using two dry etching steps as shown in Figure 1C [31,32,33]. The lithographically defined resist pattern (i,ii) is first transferred into SCD via anisotropic etching (ICP-RIE), etching few hundreds of nanometers of SCD (iii). This is followed by dry etching at an oblique angle using ion beam etching (IBE) or by using a Faraday cage inside the RIE chamber to undercut the structures (iv). Finally, the resist mask is removed (v).
- The isotropic under-etching method in which a combination of anisotropic and isotropic oxygen etching (at elevated temperature) creates free standing nano-structures [34,35,36]. A hard mask pattern (SiN) is transferred into SCD (i). A conformal coating with Al_2_O_3_ is carried out using atomic layer deposition (ii). Subsequently, the top surface of Al_2_O_3_ is dry etched (iii) to keep only the sides of structure covered. A quasi-isotropic oxygen etching at 200° C is carried out to undercut the structure (iv). Finally, the mask is removed (v).
- Si membrane hard mask transfer method where re-usable Si membranes are used as hard masks, which are patterned using mature silicon microfabrication techniques. These membranes are transferred onto diamond substrates for creating free-standing membranes via dry etching methods [37].

Heteroepitaxially grown SCD might provide an alternative route to free-standing structures, however, it is still challenging to perform high quality SCD heteroepitaxy and high-quality layers typically have millimeter thickness. Recently, Schreck et al. [3,38] reported heteroepitaxial growth of a 100 mm diameter, (100)-oriented, millimeter thick, SCD wafer on Ir/YSZ (yttria stabilized zirconia)/Si substrate via CVD. Consequently, this material system might allow up-scaling to wafer scale processing, whereas the challenge of thin membrane fabrication and free-standing structures remains. 

### 2.3. High Q-Photonic Resonators in SCD

#### 2.3.1. Photonic Crystal Resonator

**Figure 2:**
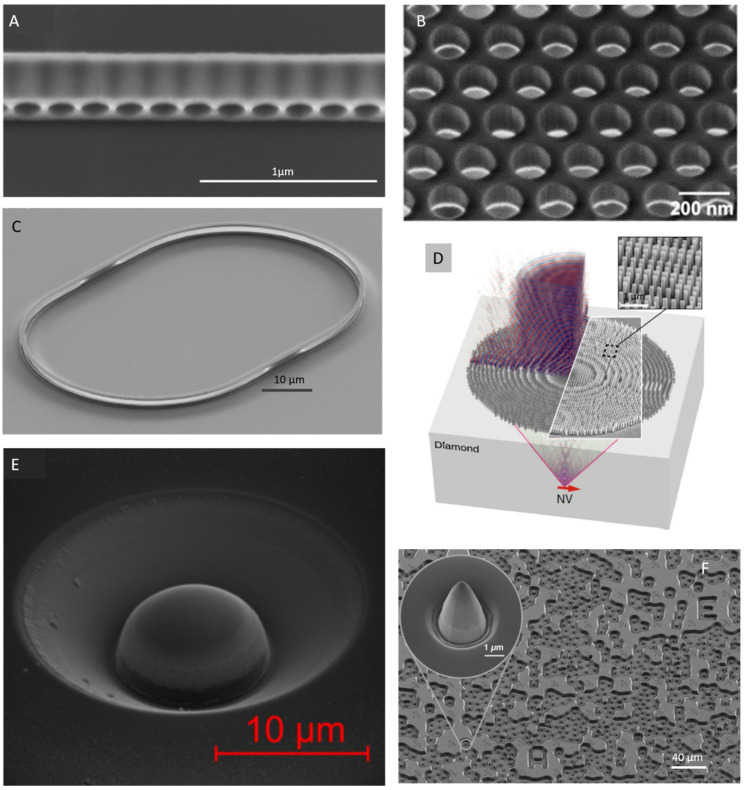
Scanning electron microscopy images (**A**–**C**) of different photonic components realized using lithography and dry etching processes: (**A**) rectangular nanobeam photonic crystal cavities (side view). Reprinted from Ref. [34], with the permission of AIP Publishing. (**B**) 2-D photonic crystals suspended in air, fabricated via quasi-isotropic undercut etching of diamond using oxygen plasma. Reprinted from Ref. [35], with the permission of AIP Publishing. (**C**) Diamond race track resonator fabricated via reactive ion beam angled etching. Image reprinted under creative commons CC-BY 4.0 from Ref. [40]. (**D**) Metalens consisting of a metasurface in which subwavelength pillars are etched into single crystal diamond substrate with naturally abundant NVs, using a combination of EBL and ICP-RIE. SEM image shown in the inset gives the zoomed-in view of the nanopillars forming the metalens. Figures reprinted from Ref. [41] under creative commons license CC BY 4.0. (**E**) SEM image of a solid immersion lens fabricated via focussed ion beam milling into single crystal HPHT diamond. In this example, the SIL incorporates native GeV center in the material. Figures reprinted from Ref. [30] under creative commons license CC BY 4.0. (**F**) Patterned diamond chip consisting of parabolic reflectors, individual one shown in the inset. Reflectors are 5 µm in height, and are irregularly arranged on the diamond chip due to NV center location dependent patterning of resist mask. Reprinted (adapted) with permission from Ref. [42]. Copyright (2018) American Chemical Society.

For quantum information, 1-dimensional nanobeams (≈200 nm thick) or 2-dimensional photonic crystal slabs with high-quality factors are advantageous. They potentially allow for large scale systems with multiple emitters per cavity coupled strongly in addition to arrays of multiple emitter-cavity nodes, photonic/spin quantum gates and quantum memories [1]. Such structures are mainly fabricated using two methods: SCD membranes thinned from bulk, and suspended SCD structures formed via angled etching [34]. For the first approach, obtained Q-factors are moderate: few thousand near the ZPL (e.g., NV ZPL). Furthermore, the yield of cavities across thinned membranes is often low due to the initial variations in thickness of the starting membranes, and thus presents a major challenge in scaling up of the fabrication. In a recent report, Mouradian et al., demonstrated quasi-isotropic oxygen under-cut etching to fabricate rectangular nanobeam cavities in SCD [34]. Due to the crystal plane dependent quasi-isotropic etching process, structures were pre-aligned with the fastest etching plane (100) to achieve rectangular cross-section of the nanobeams. The side view of the rectangular nanobeam photonic crystal cavities is illustrated in Figure 2A [34]. Rectangular nanobeam cavities have low out-of-plane scattering losses in contrast to the nanobeam cavities with triangular cross-sections. The developed fabrication process was extended to realize suspended 2-D photonic crystal (PhC) nanocavity slabs (see Figure 2B) [35]. 2-D PhC slab cavities may improve *P_F_* in comparison to 1-D PhC, as they inhibit unwanted spontaneous emission out of the cavity in the 2-D bandgap. The developed quasi-isotropic etching process is highly reproducible, with minimum process optimization requirements and enables high Qs exceeding 14,000 within 1 nm of NV^−^ ZPL (λ = 637 nm) as well as uniform cavity fabrication over an entire chip. However, controlling surface roughness of the isotropically etched structures is limited. Ref. [33] reports angled-etching of patterned diamond structures using ion beam etching instead of Faraday cage based ICP-RIE etching to overcome the limitations of non-uniform etch rates (due to the imperfections in Faraday cage mesh). These nanophotonic cavities were incorporated with SiV centers using lithographically masked implantation process and coupled with microwave coplanar waveguides (250 nm thick gold) onto the same chip to form an integrated quantum nanophotonic register. 

#### 2.3.2. Other Resonators

Other resonators such as ring resonators can be etched into suspended SCD membranes or structured into diamond-on-insulator. These structures are usually coupled with waveguides and are suitable for on-chip photonics. Microdisk resonators are generally stand-alone devices in which the spontaneous emission is out-coupled using evanescent fiber coupling and sustain ‘whispering gallery’ electromagnetic modes [1,7,43,44].

Recent reports [7,44] realized SCD membranes (≈300 nm) with GeV color centers using a combination of lift-off method and CVD. Metallic (Ge) and germanium oxide (GeO_2_) sources introduced uniformly distributed GeV centers during CVD growth and the damaged layer was etched-off using ICP-RIE. Photonic resonators (with Q ≈ 1500) such as microdisks and microrings were fabricated using a combination of EBL and ICP-RIE [7]. In another approach, high-Q/V microdisk device in SCD was realized using a combination of anisotropic and quasi-isotropic oxygen undercut etching method by Khanaliloo et al. [45].

A novel method called reactive ion beam angled etching (RIBAE) was recently developed for highly uniform and scalable processing of free-standing photonic and mechanical nano-structures [40]. RIBAE is derived from ion beam etching (IBE) or ion beam milling in which inert gases (such as Ar, Xe) used for sputtering of the target material are replaced by reactive gases such as chlorine or oxygen to extend the range of etchable materials. A race-track resonator realized in bulk SCD via RIBAE method is shown in Figure 2C. To achieve 3-D nano-structures using this method, an etch mask is patterned initially using conventional lithography techniques, followed by RIE with the sample mounted perpendicularly to the ion beam on a rotating sample stage. Subsequent etching is carried out by tilting the sample with respect to the ion beam resulting in uniform etching of the structures underneath the etch mask. The developed method yields highly uniform structures over large areas ≈ 200 mm in diameter and depends mainly on the size of the ion beam source [46].

### 2.4. Waveguides for Collection Enhancement

After discussing resonator like structures, we now turn to the waveguide structures used to modify the emission properties of color centers.

#### 2.4.1. Metalens

Metalenses are a comparably recent approach to collimate the emission from individual color centers: a metasurface composed of diamond nanopillars arranged in a regular pattern acts as an immersion lens and allows efficient coupling of color center emission to optical fibers. The diamond nanopillars constituting the metasurface are generally structured using a combination of EBL and RIE on a SCD surface [41,47]. A metasurface with subwavelength nanopillars etched into bulk SCD is shown in Figure 2D. Inset illustrates SEM image of the nanopillars [41].

#### 2.4.2. Solid Immersion Lenses (SIL)

Solid immersion lenses (SIL) consist of a hemisphere into which the color center is embedded and overcome total internal reflection. However, photon collection remains limited due to Fresnel reflection [47] which can be minimized using anti-reflective coatings. These structures allow significant improvement in photon collection efficiency in comparison to color centers in bulk diamond. Moreover, SILs do not require to position color centers close to SCD surfaces, which might limit their spin and optical properties. 

SILs are fabricated via RIE or focussed ion beam milling of hemispheres in high quality SCD. Especially for the later method, the SILs are annealed and acid cleaned to minimize crystal damage and contamination induced due to the milling process [29]. Figure 2E, shows an SEM image of a SIL fabricated via FIB milling [30].

#### 2.4.3. Parabolic Reflector

Monolithic, parabolic reflectors for broadband photon extraction from color centers were recently reported by Wan et al. [20,42]. SEM image of the patterned diamond realized using conventional EBL and RIE is shown in Figure 2F, with individual parabolic reflector shown in the inset. The reflectors are irregularly arranged on the diamond chip as the mask has been aligned with respect to individual, predefined NV centers. These parabolic reflectors represent the brightest single-photon source in diamond.

#### 2.4.4. Single Crystal Diamond Nanopillars/Nanowires

Diamond nanopillars and nanowires enhance the photon collection from color centers thus, they can be advantageous for single photon sources but also as monolithic diamond scanning probes in magnetometry. Various realizations of this geometry incorporating several types of color centers have been reported. 

**Figure 3:**
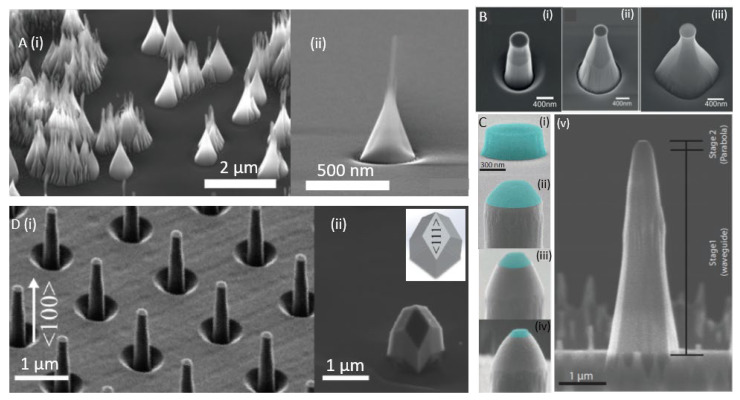
Scanning electron microscopy images of (**A**) (i) Tapered diamond nanopillars ((001)-oriented) with tip and conical base diameter of 15 nm and 200 nm patterned via ICP-RIE using silica bead masking layer over CVD grown diamond. (ii) High-resolution image of a single tapered nanopillar. Images reprinted with permission from Ref. [51] © 2020 WILEY-VCH Verlag GmbH & Co. KGaA, Weinheim; (**B**) Crystallographic orientation dependent etching of diamond to achieve nanopillars (top diameter of 350 nm and length of 1.5 µm) with tapering angles (half apex angle) of (i) 3.4°, (ii) 11.5°, (iii) 21° obtained by varying the RF power during dry etching process. Images reprinted with permission from Ref. [52] © 2018 WILEY-VCH Verlag GmbH & Co. KGaA, Weinheim; (**C**) Images depicting the fabrication process of diamond nanopillars with parabolic curvature, (i) 300 nm thick, 1 µm wide disc shaped resist (FOX-16) mask patterned via E-beam lithography, (ii) first step of dry etching (ICP-RIE) to erode the mask at the edges, have trapezoidal cross-section with base diameter of 900 µm, (iii, iv) subsequent etching with controlled erosion of resist mask to obtain parabolic diamond surface, (v) Final nanopillar consisting of a parabolic tip with a ≈ 200 nm flat end-facet and long tapered waveguide region. Figure reprinted from Ref. [20] under creative commons CC BY 4.0 license; (**D**) (i) Cylindrical diamond nanopillars (200 nm diameter and 2 µm length) fabricated using a combination of E-beam lithography and dry etching methods followed by (ii) an overgrowth step using microwave plasma enhanced CVD technique to obtain pyramidal pillars. Inset: schematic representation of the resulting pyramids with <111> crystal facet. SEM images taken at 45° angle. Figures reprinted from Ref. [53] within the open access publishing agreement of the Optical Society of America.

The first developed devices were SCD nanowires (200 nm wide and 2 µm tall) fabricated via a combination of EBL and RIE on HPHT, (100)-oriented type Ib diamond. The nanowire acts as waveguide, enhancing the collection efficiency of the photons emitted from single NV centers [48]. Nanowire/nanopillar fabrication is scalable as large arrays of pillars can be etched and color centers can be precisely positioned in the pillars. (100)-oriented SCD is mostly employed for device fabrication owing to low-cost and easy polishing. However, (100)-oriented nanopillars have limitations [1]: For maximum photon collection efficiencies, the NV’s dipoles should be perpendicular to the nanopillar axis. However, in (100)-oriented nanopillars, the NV’s dipoles form an oblique angle with the pillar axis due to the aligning of NV center orientation with one of the four equivalent <111> crystal directions. Recent availability of high-purity, high-quality (111)-oriented CVD grown SCD opened the possibility to fabricate (111) oriented pillars [49]. In this material, optimal alignment of NVs is reached as the NV’s preferential alignment along the growth direction can be harnessed [50]. Some examples of nanopillars/nanowires realized are illustrated in Figure 3A–D.

We summarize recent realizations of nanopillars and the respective fabrication methods in Table 1. The most common method involves pattern definition via EBL followed by highly directional etching using ICP-RIE. Alternative methods such as using silica beads as masks to minimize the dependence on lithography systems have been adapted [51]. In Figure 3A, tapered pillars obtained using this method are shown. However, the pillar density is challenging to control in this approach. 

Nanopillars have been functionalized with a variety of color centers and various methods for color center creation are used: In addition to conventional ion implantation (before pillar fabrication) focused beams of ions allow for precise positioning (lateral and depth) into pre-structured nanopillars. For instance, Marseglia et al. [54], recently reported scalable fabrication of SCD nanowire arrays (350 nm, 650 nm top and bottom diameter and height of 1.2 µm) via EBL and ICP-RIE followed by implantation of single or multiple SiV^−^s in individual nanowires using focused ion beams. In another approach, a homoepitaxial, 100 nm thick SCD layer with SiV^−^s on high purity type IIa diamond was patterned using EBL and RIE. The realized nanopillars host single SiV centers with high spectral stability [55]. In a similar approach, pre-structured SCD pillar were coated with spin-on-glass (perhydropolysilazane) and homoepitaxially overgrown via CVD method, to obtain localized ensembles of SiV^−^s in the overgrown part of nanopillars [56]. Creating color centers during growth potentially enhances their spectral stability as well as spin properties by avoiding implantation induced damage (see also Section 2.5) [55].

#### 2.4.5. Diamond Inverted Nano-Cone (DINC) 

**Figure 4:**
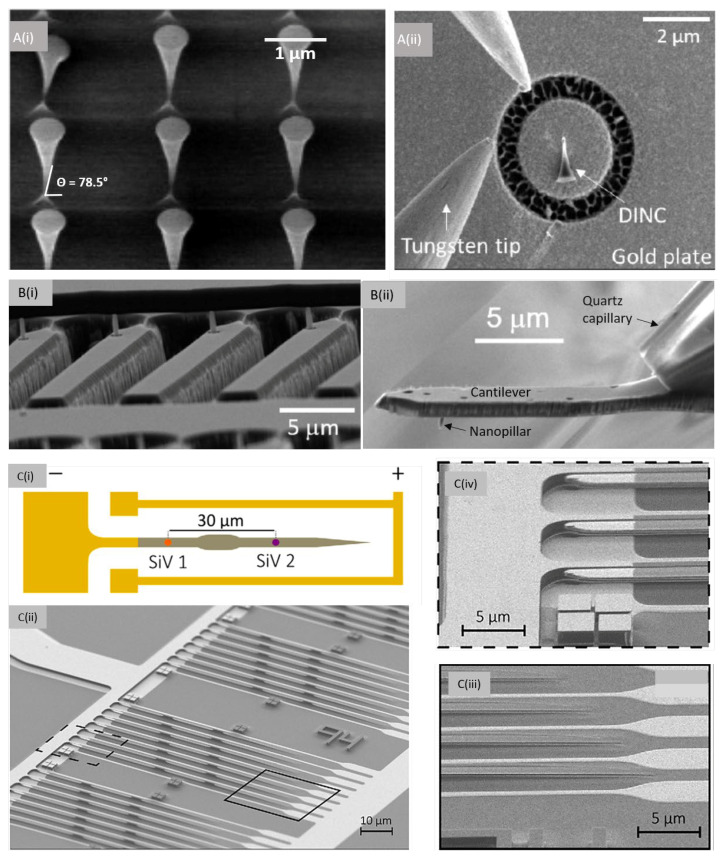
SEM images of (**A**) (i) Diamond inverted nano-cones (DINC) realized via a combination of E-beam lithography and angled dry etching (ICP-RIE) using a conical faraday cage. (ii) Transfer of DINC onto a gold target plate using tungsten tips for coupling of DINCs to other photonic components. Reprinted (adapted) with permission from Ref. [58]. Copyright (2020) American Chemical Society. (**B**) (i) An array of monolithic diamond scanning probes fabricated using multiple E-beam lithography and dry etching processes. The shown nanoprobes required back side diamond etching for release of the devices. (ii) Final released device consisting of a cantilever with nanopillar, attached to a quartz capillary which acts as a holder for mounting the device to a scanning probe microscope. Images reprinted from Ref. [59] under creative commons CC BY 4.0 license. (**C**) (i) Schematic representation of a diamond nanophotonic device where the gray region represents the diamond waveguide implanted with SiV centers at defined locations, while the yellow stripes represent the electrodes to form a capacitor. On application of bias voltage between the device (-ve terminal) and the bottom (+ve terminal), the doubly clamped cantilever is deflected and results in localized tuning of the color center. (ii) SEM image of the photonic device realized using a combination of E-beam lithography and angled etching technique of diamond substrate. Subsequent lithography steps were carried out to implant precisely implant Si^−^ ions (to form SiV centers) and lift-off process for electrode definition. (iii) Capacitor plates defined on and below the devices. (iv) Tapered diamond to efficiently extract photons from waveguides. Images reprinted from Ref. [39] under creative commons license CC BY 4.0.

A novel waveguide type nano-structure allowing for efficient photon collection was recently reported by Jeon et al.: inverted nano-cones (DINCs) were realized in high quality SCD [58]. NV centers (depth of NVs approx. 200 nm) were created via ion implantation prior to fabricating DINCs using EBL and two step RIE. First, anisotropic, vertical O_2_ plasma etching was carried out using a silicon nitride hard mask, to control the height of the nano-cone. Subsequently, angled dry etching leads to the conical shape of DINCs. To allow circularly symmetric, smooth etching of DINCs, a cone shaped Faraday cage is used. Figure 4A(i) shows the DINC’s with top and bottom diameters of 550 nm and 50 nm, respectively. The inverted taper angle of the DINCs is 78.5 degrees. Because of ion channeling, the created NV centers have a wide depth distribution within the DINC’s. Consequently, statistical analysis of photon collection efficiency of DINCs, conventional diamond nanopillars and bulk substrates is necessary to evaluate the enhancement. The DINCs showed enhanced photon collection efficiencies compared to conventional diamond nanopillars. Moreover, DINCs are easily detachable from the substrate owing to the small cross-sectional area at the bottom and relocated to other substrates with high precision. Figure 4A(ii) illustrates the transfer of a single DINC onto Au chip using a tungsten tip. 

### 2.5. Scanning Probes Based on Color Centers in Diamond Nano-Structures

NV center based sensors offer impressive sensing capabilities for magnetic and electric fields [2,60]. To allow for nanoscale spatial resolution, it is important to bring the NV center and the sample of interest as close as 100 nm or less. This requires using a scanning probe structure. Table 2 summarizes the approaches to create such structures with scannable NV centers including attachment of diamond nanocrystals to conventional AFM tips and structuring of monolithic SCD scanning probes. In an alternative approach, the sample is attached to the cantilever of a commercial scanning probe microscope and scanned over an NV embedded ≈ 5 nm below an SCD surface and is equivalent to scanning of diamond probes over the sample surface [61,62]. Ernst et al. [63] recently demonstrated another method of using planar SCD (50 µm thick) with single NV centers as scanning probes for imaging planar samples, which required highly precise alignment (radial) and proximity control between sensor and sample. While initial scanning probes relied on grafting color center containing diamond nanocrystals to AFM tips, the focus recently shifted to realization of top-down fabricated SCD nanoprobes [20,60]. They have several advantages: robust and usable in harsh environments (e.g., cryogenic temperatures), not limited by optical bleaching and excess spin dephasing and the ability to tailor the shape of the diamond tip to enhance photon collection [20].

All SCD scanning probes are fabricated using multiple steps of EBL and RIE processes. An example of an array of fabricated nanoprobes is illustrated in Figure 4B(i). The probes are mounted to a holder e.g., a capillary for integration into an AFM system as shown in 4B(ii). Most scanning probes developed till date comprise of truncated cones with blunt, circular end-facet of ~200 nm diameter [53]. Though such tips have beneficial photonic properties, their blunt facet prevents obtaining high resolution AFM imaging in parallel to e.g., magnetometry. Moreover, the blunt tips require precise angular alignment to not create an additional, unwanted distance between color center and sample.

Tailoring the scanning probe geometry can enhance photon collection, AFM capability and spatial resolution. Nanopillars with large taper angles obtained via plane dependent dry etching enhance photon collection due to the combined effect of optical wave guiding and adiabatic changes of the effective refractive index [52]. Figure 3A,B show pillars with various taper angles. Such tapered pillars also improve the mechanical strength of the probe. In another approach, nanopillars with parabolic curvature (200 nm flat-end facet) and long tapering waveguide section achieve 5-fold increment in photon collection efficiency from a near surface NV [20]. The parabolic shape is achieved via a two-step ICP-RIE process shown in Figure 3C, in which the gas mixture is changed to obtain a parabolic end facet with long tapering waveguide section. Giese et al. [64] recently reported high aspect-ratio (>60), triangular diamond nanobeams fabricated via Faraday cage angled etching, integrated into an AFM setup. This approach has the advantage that it omits the preparation of thin membranes. Integrating NVs at the extremity of such nanobeams allows high photon collection efficiency along with high scanning resolution of structures with steep sidewalls. 

One route to optimized nanopillar scanning probes might be to combine top-down and bottom-up methods to develop pyramidal probes [53]. To this end, Batzer et al. used cylindrical nanopillars (diameter = 200 nm and length = 2 µm) fabricated using EBL and ICP-RIE on (100)-oriented SCD [65]. Figure 3D(i), shows an image of those cylindrical pillars. The pillars were then overgrown using microwave plasma enhanced CVD, creating smooth and sharp pyramidal tips with tip radii of 10 nm (see Figure 3D(ii)). Such sharp pyramidal tips with near surface color centers are promising for high-resolution AFM imaging, combined with enhanced spatial resolution color center based sensing, photonic properties and long spin coherence time. Moreover, also for scanning probes, recently the more advantageous (111) diamond orientation has been used [66].

### 2.6. General Aspects of Color Center Creation, Surface Termination and Tuning

Before proceeding to nanomechanical structures, we here summarize some aspects of color center creation and surface termination, relevant for the discussed nano-structures. For various quantum applications, shallow NV centers less than roughly 50 nm below the surface are necessary. Single NVs are preferred for high spatial resolution sensing, e.g., scanning probe techniques, however, the fluorescence signal is weak. In contrast, ensembles of NVs can give better sensitivity and fast imaging of a large area of interest with reduced spatial resolution [50,67]. Shallow NV centers are created using low energy ion implantation and delta-doping often combined with electron irradiation, or implantation of helium ions [68,69]. Although these methods have high positioning accuracy, they are limited in conversion efficiency (only a few % or less) of implanted nitrogen creates NV centers (yield) [70]. Part of the created vacancies are lost in recombination with carbon interstitials, vacancy complex formation (di-vacancies, vacancy chains) or diffusion to the diamond surface [71]. Additionally, shallow, implanted NVs have relatively short spin coherence time (T_2_~10 µs in comparison to 600 µs for NVs in bulk), and exhibit instability of the negatively charged state of NV^−^ [72,73]. It is mainly attributed to the implantation induced damage formed in the diamond lattice in close vicinity to the implanted NV centers, which is not fully eliminated post thermal annealing.

Thus, in addition to the fabrication of nano-structures, optimized methods to create shallow color centers are central for quantum technologies. These include optimization of the thermal annealing condition after nitrogen implantation, treatment in soft oxygen plasma and additional annealing steps in oxygen environment. Annealing in oxygen environment at 450 °C enhances the surface ordering at the atomic scale thereby enhancing NV coherence, which is limited below tri-acid cleaned surfaces as the oxygen groups are highly disordered [72]. Moreover, oxygen annealing stabilizes the negatively charged state of shallow NVs [73]. Significant improvement in T_2_ (10-fold) was achieved using a thin, *p*-doped diamond layer (e.g., boron) epitaxially grown on ultrapure diamond, prior to nitrogen implantation. This approach charges vacancies and prevents vacancy cluster formation during annealing, consequently enhancing the NV formation yield [70]. In another approach, Herbschleb et al. reported usage of high-quality *n*-doped diamond with phosphorus atoms to improve NV based sensing [74]. The highest NV center yield (75%) with enhanced T_2_ was reported in sulphur-doped diamond ((100)-CVD grown) which also provides highest NV^−^ stability. 

The influence of diamond surface termination via multiple atoms such as oxygen, hydrogen, fluorine, silicon and nitrogen on the charge stability of shallow implanted NV^−^s has been studied [72,73]. It has been found that the surface termination with negative electron affinities (NEA) such as on termination with hydrogen or silicon, results in instability of the charge state of shallow NVs. On the other hand, termination of surfaces with positive electron affinities (PEA) for instance with oxygen or fluorine, enhances the stability of NV^−^s. In a recent report, Kawai et al. investigated the influence of Si and N_2_ termination on the stability of shallow implanted NV^−^s. The termination with Si and N_2_ was carried out by silicon evaporation at high temperature (950 °C) and nitrogen radical beam exposure, respectively on high purity diamond grown over (001)-oriented diamond substrate. It was found that Si termination of the surface yielded unstable NV^−^s, while nitrogen terminated surfaces (full *n* or N/H) stabilized NV^−^s with positive surface electron affinity and spin properties (e.g., coherence time) comparable to that of oxygen terminated surfaces. 

In addition to charge state instabilities and reduced coherence, especially color centers in nano-structures can show a spread in transition frequencies which makes their use especially in quantum information challenging. Recently, nano-structures have been presented that directly implement local tuning of color center transitions via strain. Figure 4C(i) shows such structures, in which the grey region represents a waveguide section with SiV centers implanted at defined locations, while the yellow stripes represent metallic contacts forming a capacitor [39]. The doubly clamped cantilever is deflected on application of a bias voltage between the device (-ve terminal) and the bottom (+ve terminal) and leads to localized strain tuning of the SiV centers. Figure 4C(ii) shows an SEM image of such a device. These devices were realized using a combination of EBL and angled etching. Further lithography processes were implemented to precisely implant Si^−^ ions (to form SiV centers) and to define metallic electrodes via a lift-off process. The inset Ciii shows the waveguide taper for efficient extraction of photons, while Civ represents the capacitor plates patterned on and below the devices.

## 3. Nanomechanical Components

Nanomechanical components such as cantilevers have a very small mass and are highly responsive to changes in their local environment. Applications of nanomechanical components include scanning probe magnetic and atomic force microscopy that yields attonewton level sensitivities as well as the detection of the mass of single molecules and proteins in real-time [1,77]. 

The minimum detectable force *F_min_* using a nanomechanical cantilever is limited by thermomechanical noise which is governed by dissipation of mechanical energy and absolute temperature (*T*) [1]:

(3) $$F_{min}=\;\sqrt{\frac{2k_{B}TBk_{c}}{\pi f_{c}Q_{m}}}$$

where *k_c_*, *f_c_*, *k_B_* represent the spring constant, resonant frequency, and Boltzmann constant respectively. *Q_m_* represents the mechanical quality factor and characterizes the bandwidth of the resonator (i.e., *B*) with respect to *f_c_*. High *Q_m_* values indicate low rates of loss of mechanical energy. For a rectangular cantilever with no-internal stress, *F_min_* can be described using thickness (*t*), width (*w*), length (*l*), mass density (*ρ*) and Youngs modulus (*E*) of cantilever [1]:

(4) $$F_{min}=\;\sqrt{\frac{wt^{2}k_{B}TB}{lQ_{m}}\;\sqrt{E\rho }}$$

To enhance the sensitivity of the cantilever, it is required to decrease the mass and keep the cantilever narrow, thin and long. Several efforts have been made to develop thinner cantilevers with lower *k_c_*’s in conventional materials such as Si or by fabricating doubly clamped beams. However, reducing the thickness of nanocantilevers leads to decrease in *Q_m_* due to increase in the surface friction by surface defects. This issue is overcome by using cryogenic temperatures which limits the practical applications of such resonators.

The huge interest in realizing nanomechanical components based on diamond is due to its outstanding mechanical and thermal properties and the possibility to embed spin-carrying color centers into nanomechanical components. Diamond’s record high bulk Young modulus provides high mechanical resonance frequencies and low mechanical losses. Also, its high thermal conductivity and low thermal expansion coefficient allows high dissipation of heat. Color centers enable optical readout of nanoresonator motion with the capability of magnetic sensing and to implement optomechanics on the same platform. These properties are retained at room temperature and with the known biocompatibility of diamond, nanoresonators are suitable for sensitive detection of living cells/tissues.

Diamond nanoresonators are fabricated using the top-down methods described in Section 2 for photonic components. Commercially available diamond membranes are transferred to carrier substrates for easy handling and patterned using a combination of lithography and RIE. In the following subsections, we briefly describe advanced diamond nanoresonators realized in recent years and discuss their advantages. 

### 3.1. Nanocantilevers

Diamond nanocantilevers and nanobeams are used for ultrahigh force sensitivity. Nanocantilevers are clamped on one end while the beams are clamped on both the ends. Commercial diamond substrates are pre-processed using RIE and polishing to achieve low surface roughness (<5 nm) and high thickness uniformity (better than 1 µm variation over mm sized sample) to reliably realize cantilever/nanobeam resonators via lithography and RIE.

**Figure 5:**
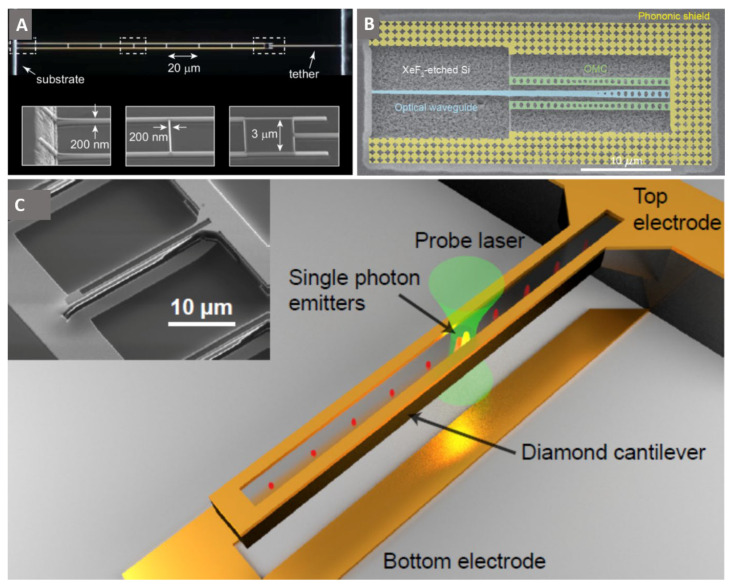
(**A**) Optical micrograph of a single crystal diamond nanoladder cantilever (150 µm long) realized using a combination of E-beam lithography and dry etching processes. Each nanoladder device consists of two parallel nanowires (200 nm wide) connected via rugs at every 20 µm distance. The zoomed in view of the base, middle and tip regions of the ladder is shown in the insets. Reprinted (adapted) with permission from Ref. [12]. Copyright (2018) American Chemical Society. (**B**) SEM image of a pair diamond optomechanical crystals on either side of diamond optical waveguide realized using diamond on insulator technique. The optomechanical crystal consists of a rectangular cross-section, single crystal diamond nanobeam with a one-dimensional array of ellipses etched along its length. Image reprinted with permission from Ref. [28] copyright IOP publishing, Reproduced with permission. All rights reserved. (**C**) Schematic representation of a diamond microcantilever realized using a combination of E-beam lithography and angled etching technique followed by electrode definition using e-beam lithography and lift-off process. The color centers (GeV) are precisely implanted (lateral accuracy > 50 nm) using focussed ion beam implantation technique. Application of a bias voltage between the top and bottom electrodes causes strain in the cantilever. Using a probe laser, multiple color centers (GeVs) can be simultaneously optically accessed. SEM image of the diamond microcantilever with dark and light regions representing the diamond and electrodes is shown in the inset. Reprinted with permission from Ref. [79]. Copyright (2018) by the American Physical Society.

Top-down fabricated nano-resonators have low device-device variability. According to Equation (3) (*F_min_* the sensitivity of nanomechanical sensors is limited by thermomechanical force noise, which is reduced for structures with low spring constant *k_c_* and mass, m. Tao et al. developed rectangular cantilevers with high *Q_m_* (>10^6^ at mK temperatures) by fabrication using robust diamond on insulator or quartz bonded substrates [77]. Additionally, functionalization of cantilever surfaces with oxygen and fluorine groups has shown to positively affect the surface friction (decrease in surface friction due to cleaner and better defined surface groups) and thus influence the *Q_m_* [77,78]. In a recent report by Eichler et al. [12], nanoladder configuration cantilevers are used to decrease the cross-section while maintaining the cantilever length thereby keeping the *k_c_* and m low. The nanoladder cantilever consists of two parallel nanowires (200 nm wide and 150 µm long) connected by struts (200 nm wide, 3 µm long) along their length and was realized using a combination of EBL and ICP-RIE. Figure 5A shows the nanoladder cantilever with zoomed in-view of individual regions (base, middle and tip regions) in the insets. Highest *Q_m_*-factors measured with the realized nanoladder cantilever at mK temperatures were 162,000.

### 3.2. Nanowires

Diamond nanowires integrated with Si/diamond cantilever are used for sensitive force detection in scanning force microscopy. Owing to its low dielectric constant, diamond has low non-contact friction which normally deteriorates force sensitivities when the tip is too close to the surface [1]. The realization of nanowires (with different diameters and lengths) for force sensing applications is carried out by methods described earlier for fabrication of nanopillars.

## 4. Optical and Mechanical Hybrid Systems

Hybrid mechanical systems in which quantum elements such as qubits are coupled to mechanical oscillators attracted significant attention in recent years due to their potential in addressing key issues in quantum information processing, communication and control. Cady et al. recently realized a diamond optomechanical crystal (OMC) with embedded NV centers via the diamond on insulator technique [28]. The OMC design consists of an SCD nanobeam with a rectangular cross-section in which a one-dimensional array of ellipses is etched via ICP-RIE. Figure 5B shows a pair of SCD OMCs on either side of an SCD optical waveguide, surrounded by a phononic shield. The NV center interacts with the mechanical motion of the OMC through strain in the crystal. The light emitted by the NV center is measured using a confocal microscope and the OMC is probed through coupling to the adjacent optical waveguide. The rectangular cross-section OMCs are promising for hybrid NV-mechanical systems owing to the z-symmetric strain profile of the fundamental breathing acoustic mode with the feasibility to realize 2-dimensional phononic shields, paving the way for high mechanical quality factors. Maximum Quality factors of 42000 were achieved with the realized OMCs, which further need to be improved (to reach Q~10^6^) in addition to NV center spin coherence to enhance coupling between an NV center spin and mechanical motion of OMC.

Color centers in diamond are important components of optical quantum technologies, however, photons from different emitters are often spectrally distinguishable due to the complex mesoscopic environments of the color centers. Strain-based spectral alignment of two solid-state single photon emitters was achieved by Maity et al., using the strain gradient in diamond microcantilevers [79]. The diamond microcantilever in Figure 5C was fabricated using a combination of EBL and angled-etching. Subsequently, EBL and lift-off were implemented to define the electrodes. GeV color centers are implanted precisely via focused ion beam implantation. An SEM image of the microcantilever is shown in the inset with dark and light regions representing the diamond and the electrodes, respectively. The cantilever is strained by a bias voltage between the top and bottom electrodes and multiple GeV’s can be simultaneously accessed optically using a probe laser. The developed mechanism paves the way to realize identical quantum emitters for on-chip integrated quantum systems.

Moreover, conversion between different colors of light is important for coupling quantum optical technologies which have different optical wavelengths. Optomechanical conversion of wavelengths and amplification has been recently demonstrate in SCD microdisk cavities by Mitchell et al. [80]. The realized devices are promising for converting the emission of diamond color centers to telecommunication wavelengths for applications in quantum networks.

## 5. Other Diamond Nano-Structures 

### 5.1. Diamond Mirrors

**Figure 6:**
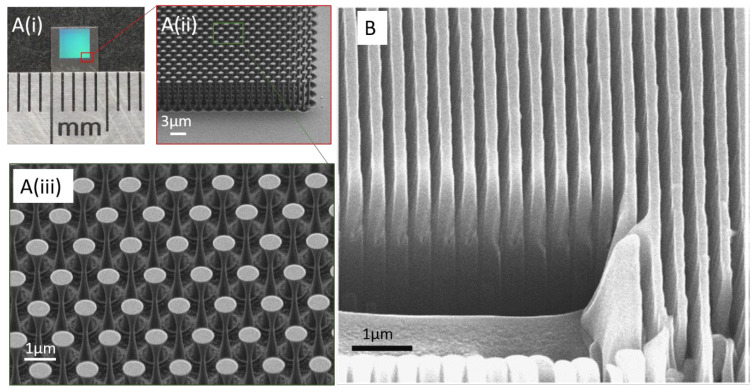
(**A**) (i) Optical microscope image of diamond mirrors (on 4.2 mm × 4.2 mm diamond chip) fabricated via reactive ion beam angled etching. Each division on the ruler measures 1 mm. A (ii) SEM image of the diamond mirror taken at 60° angle with zoomed in view A (iii) showing golf tee features of the mirror taken at 40° angle. Reprinted with permission from the author from Ref. [46]. (**B**) SEM image of 400 nm pitch diamond nanogratings fabricated via a combination of interferometric lithography and dry etching. Figure reprinted from Ref. [81] under creative commons CC BY 4.0 license.

Optical components such as mirrors which withstand extremely high laser powers are desired to steer the laser to the target location in applications varying from laser manufacturing, semiconductor industry and medicine [46]. Conventional mirrors consist of multilayer coatings of thin dielectric layers of different refractive index and thickness. Defects and imperfections in the multilayer dielectric coatings lead to absorption of laser energy at high powers, thereby causing thermal stress and permanent damage. These limitations were overcome by realizing monolithic diamond mirrors which are not limited by thermally induced damage. Diamond mirrors created by etching nano-structures into diamond surfaces, exhibited favorable optical properties with extremely high thermal conductivity. An optical image of diamond mirrors (on 4.2 mm × 4.2 mm diamond chip) fabricated using a combination of EBL and RIBAE is illustrated in Figure 6A(i). Each division on the ruler measures 1 mm. In Figure 6A(ii), SEM image of the diamond mirror taken at 60° angle is shown with zoomed in view (iii) depicting golf tee features of the mirror taken at 40° angle. The realized diamond mirrors show high reflectivity (>98%) and are capable of withstanding extremely high laser powers.

### 5.2. Diamond Nanogratings

Sensors based on NV centers are promising for low volume NMR spectroscopy, but are limited in sensitivity [81]. Kehayias et al. recently reported a method to enhance the surface area of such sensors via nano-structuring of dense high-aspect-ratio nanogratings. In Figure 6B, an SEM image of such diamond nanogratings with 400 nm pitch realized using a combination of interferometric lithography and RIE is shown. The sidewalls of the nanogratings are doped with NV centers few nanometers below the diamond surface and are deployed for NMR spectroscopy in 1 pL volume of solution placed adjacent to nanograting grooves at room temperature. Using the realized diamond nanogratings, approximately two orders of magnitude improvement in concentration sensitivity from previously reported NV and picolitre NMR studies was obtained.

## 6. Conclusions and Outlook

In this review, we summarized advanced nano-structuring methods for diamond. The different top-down fabrication methods mainly comprising of lithography and dry-etching processes are described. In recent years, various novel configurations of nano-structures with applications varying from photonics, mechanics, optomechanics, nanoelectronics, mirrors etc. were realized and are reviewed here. The methods for precise and controlled creation of color centers such as NVs or group-IV defects for specific applications are also discussed.

Though the advancement in nanofabrication methods resulted in novel nano-structures with superior photonic or/and mechanical properties there are certain limitations which are hindering up-scaling the processes. The growth of wafer-scale, high quality, single crystal diamond is limited, while nano-structuring of individual diamond substrates often results in device-to-device variability. Optimal processes are still being developed: e.g., the commonly used e-beam resist HSQ adheres poorly to diamond and thus suitable adhesion promoting layers such as Si or SiO_2_ are required [41,59]. Additionally, HSQ is highly sensitive to oxygen in air with low shelf life, leading to very limited stability after coating. This limits batch processing of diamond nano-structures. Moreover, selectivity of HSQ based masks to oxygen plasma etching is imperfect, and results in variation in device cross-sections which affects the photonic/mechanical properties. These issues are mitigated by using metal based hard masks (such as Al) which require additional pre-/post-processing steps. Other alternative approaches will require HSQ-like resists with better adhesion and stability to minimize the processing time, improve the device-to device reproducibility and up-scale the fabrication process.

The novel nanophotonic/nanomechanical devices realized via top-down methods might be affected by plasma induced damage. Furthermore, implantation based methods to create color centers create defects which degrade the properties of the final devices. These issues are inherent to top-down fabrication methods and need to be addressed by usage of alternative plasma mixtures, surface treatment methods, etc. [4]. Other methods to overcome this include combination of both bottom-up and top-down methods to realize diamond nano-structures with enhanced surface and optical properties [53].

In summary, while nanodevices in diamond have tremendously improved in recent years, large scale, low-cost availability of high-quality diamond, nano-structuring with minimum influence on device properties remain challenging to make these devices suitable for a broad range of applications in the fields of quantum information to sensing. Additionally, the realized devices should be easy to handle and integrate onto other photonic/mechanical platforms to develop multi-functional, on-chip hybrid systems.

## Figures and Tables

**Table 1:** Short description of different fabrication methods developed for nanowire/nanopillar realization.

Methods Used	Fabrication Method	Properties/Comments	References
Type/Dimensions			
SCD nanopillars with single tin vacancy center: 200 nm diameter; 500 nm height	Preimplantation with Sn atoms SnV^−^ depth of 90 nm EBL & ICP-RIE to fabricate pillar pattern, Silicon nitride (Si_x_N_y_) as hard mask	Obtained narrow emission linewidths (<250 MHz) of single SnV^−^	[57]
SCD nanopillar: Tapered with tip & conical base diameter of 15 nm & 200 nm	(100) & (111)-oriented CVD grown diamond Dilute Silica bead solution spread over diamond used as a mask for ICP-RIE	Two step etching process: 1st to obtain nanopillars & 2nd to remove any organic/amorphous layer Density of nanopillars difficult to control	[51]
SCD Nanowires with single SiV^−^ center: 350 nm top diameter, 650 nm bottom diameter & height of 1.2 μm	High purity, type IIa, HPHT SCD Nanowires created via EBL & ICP-RIE Si ions implanted (AuSbSi source) via nanoimplanter at a penetration depth of 120 nm	Scalable fabrication method Nanowires with single or multiple SiV^−^s Incorporation of single SiV^−^ in nanowires yields higher light coupling efficiency (10 times) in comparison to single SiV^−^ center in bulk diamond.	[54]
Overgrown SCD pillars with SiV ensembles: Tapered with diameter varying from 200–1000 nm & height of 1 µm	(100)-oriented SCD EBL and lift-off of deposited Al hard mask, ICP-RIE. Homoepitaxial overgrowth via CVD after spin coating with spin-on-glass	Ensembles of SiV^−^s localised in the overgrown pillars Shape of overgrown part varies with initial diameter of the pillars: cubic for 500 nm & 1000 nm, pyramidal for 200 nm pillar	[56]
SCD nanopillars with single SiV centers: 135–170 nm in diameter, 200 nm in height	High purity, type IIa SCD 100 nm thick homoepitaxial SCD layer containing in-situ created SiV centers Pattern HSQ layer via EBL & RIE	Optimized fabrication process to obtain single SiV per pillar High spectral stability of CVD grown SiV centers	[55]

**Table 2:** Examples of different shapes/assemblies of diamond for scanning probe magnetometry applications.

Properties	Shape & Dimensions of Scanning Probe	Fabrication Method	Comments	References
Type of Scanning Probe				
All diamond scanning probe (100)-oriented diamond. NV orientation and magnetic field sensing is along an axis tilted by 54.7° from nanopillar direction.	Nanopillar: diameter = 350 nm; L= 3.5 µm Cantilever probes: L= 125 µm, W = H = 50 µm	EBL & ICP-RIE for nanopillar structuring with 10 nm Ti as adhesion layer on diamond; UVL for cantilever probe definition; deep etch with 400 nm Ti as an etch mask. NV depth defined using ion implantation.	Robust, simple fab. process for 52 probes on 2 mm × 4 mm substrates Integrated RF components on the cantilever probes	[19]
	Triangular shaped beam: L = 30 µm, W = 450 nm	EBL for patterning with Ni as protective mask for structuring diamond; Faraday cage used for ICP-RIE to obtain high aspect-ratio triangular shaped beam.	High aspect-ratio of tip suitable for scanning of structures with steep sidewalls 2000 devices possible on 4 mm × 4 mm substrates Location of NVs not defined	[64]
	Pillar with parabolic curvature: 200 nm flat-end facet, long tapering waveguide section	Cantilever & pillar definition using EBL followed by ICP-RIE etching. A deep etch is carried out for releasing the diamond in the end.	Parabolic design yields high photon collection efficiency from a near surface NV.	[20]
	Diamond bonded on oxidized Si. Pillar: 200 nm diameter, 1 µm length; cantilever: 150 × 20 × 3 μm; Array of pillars (1 µm pitch) on each cantilever	Pillar structuring using NIL with Ti hard mask and O_2_ etching. The cantilevers released using deep RIE through Si substrate. Single NV per pillar at 15–20 nm depth.	100 cantilevers on 2 mm × 2 mm diamond. The unconventional AFM cantilever with multiple tips (pillars) favours the probability of finding shallow NV pillar with favourable coherence properties Wide-field imaging possible for large areas to obtain vector field magnetometry	[18]
	Nanopillar: 200 nm diameter	5 µm thick diamond membrane creation using shadow mask & RIE; EBL & RIE for definition of nanopillar & cantilever with 5 nm Ti as adhesion layer used for HSQ on diamond; and pre-patterned alignment marks using EBL	No ICP used, only RIE Fabrication of around 1000 cantilevers possible on 2 mm × 2 mm sample	[75]
Bulk diamond with NV centre for imaging sample scanned on top	Single NV centers embedded 5 nm below type IIa diamond membrane of 30 µm thickness	Shallow implantation of single NV centers at 2.5 keV using ^15^N^+^ ions	Only possible if sample can be attached to cantilever	[61]
Diamond nanocrystals with single NV center attached to conventional AFM tips	Nanodiamonds (20 nm) with single NV grafted on AFM tips	Diamond nanocrystals synthesized by milling of type 1b HPHT diamond crystals with high N_2_ content.	Limited in sensing performance due their short spin coherence times	[76]
